# Supplementary material for: Exercise induces cerebral VEGF and angiogenesis via the lactate receptor HCAR1
Source: Nat Commun. 2017 May 23;8:15557. doi: 10.1038/ncomms15557 (PMC5457513; doi:10.1038/ncomms15557)
Supplement: Supplementary Information — Supplementary Figures, Supplementary Tables and Supplementary References. [file ncomms15557-s1.pdf]

Supplementary Information

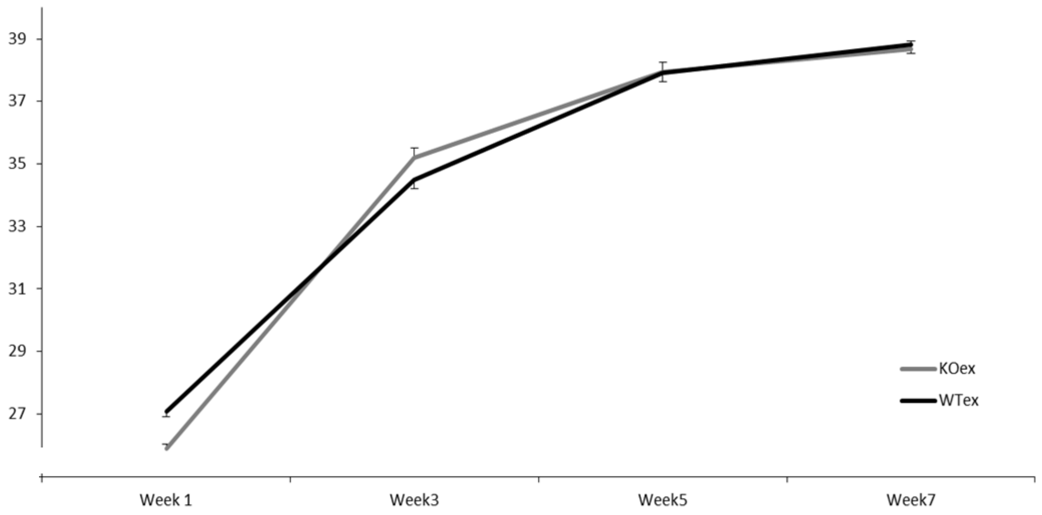

Supplementary Figure 1 | Physical performance in maximum test. The maximum running speed of the mice (m/min) in the maximal exercise capacity test (MECT) was not affected by the genotype. It reached a plateau by week 7 of exercise training. The data are presented as mean ± s.e.m., n = 20 mice for KO (8M/12F), n = 21 mice for WT (10M/11F).

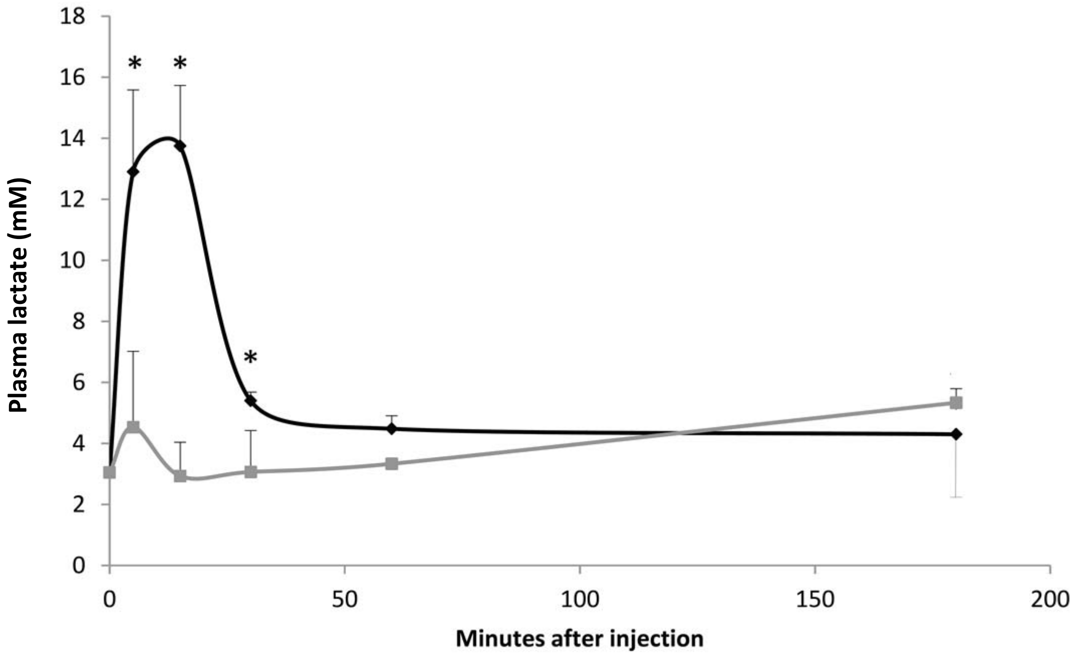

Supplementary Figure 2 | Timecourse of plasma lactate after subcutaneous injection. Plasma lactate levels (mmoles/L) increased quickly after a single subcutaneous dose of sodium L-lactate 2.5 g/kg (250 mg/ml, pH 7.4) (black line), but not after injection of the same volume of phosphate buffered saline (grey line). The lactate concentration peak was between 5 and 15 minutes after the injection. The data are presented as mean ± s.d., n= 5 mice for each time point for lactate treated mice and n=3 mice per time point for saline treated mice. The basal lactate concentration (no injection = "0 minutes") was 3.05 ± 0.72 mM (n = 4). (In this pilot experiment the injected dose was slightly higher than in the main experiment, 2.0 g/kg.) *, time points at which plasma lactate levels were significantly higher in mice treated with lactate than in mice treated with the same volume of saline; $P < 0.05$ (Chi square test; Minitab).

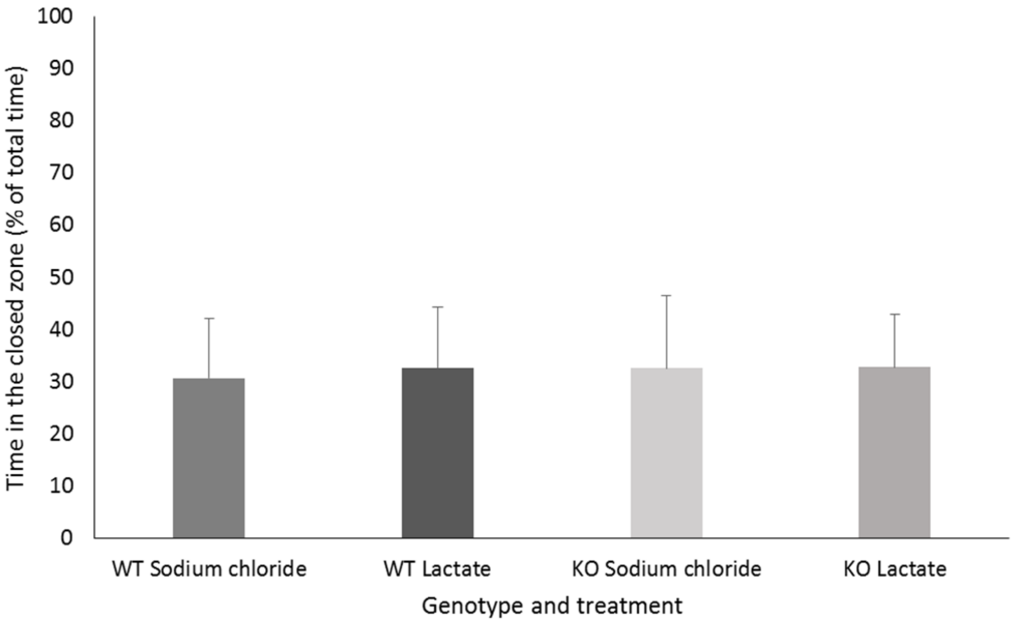

Supplementary Figure 3 | No sign of anxiety attack after lactate injections. At 15 min after the first injection of lactate, the mice were tested in the elevated zero maze[1]. The time spent in the closed zone (a correlate of anxiety) was the same in all experimental groups. Data are presented as mean ± s.d. of 14-22 mice in each group with a similar gender (M/F) distribution: WT sodium chloride 8M/6F, WT lactate 10M/12F, KO sodium chloride 11M/8F, KO lactate 12M/9F. The frequency of "head-dips" outside the edge of the open zones (negatively correlated with anxiety) was also equal in the groups (not shown). Analysis of variance (ANOVA), $P = 0.96$.

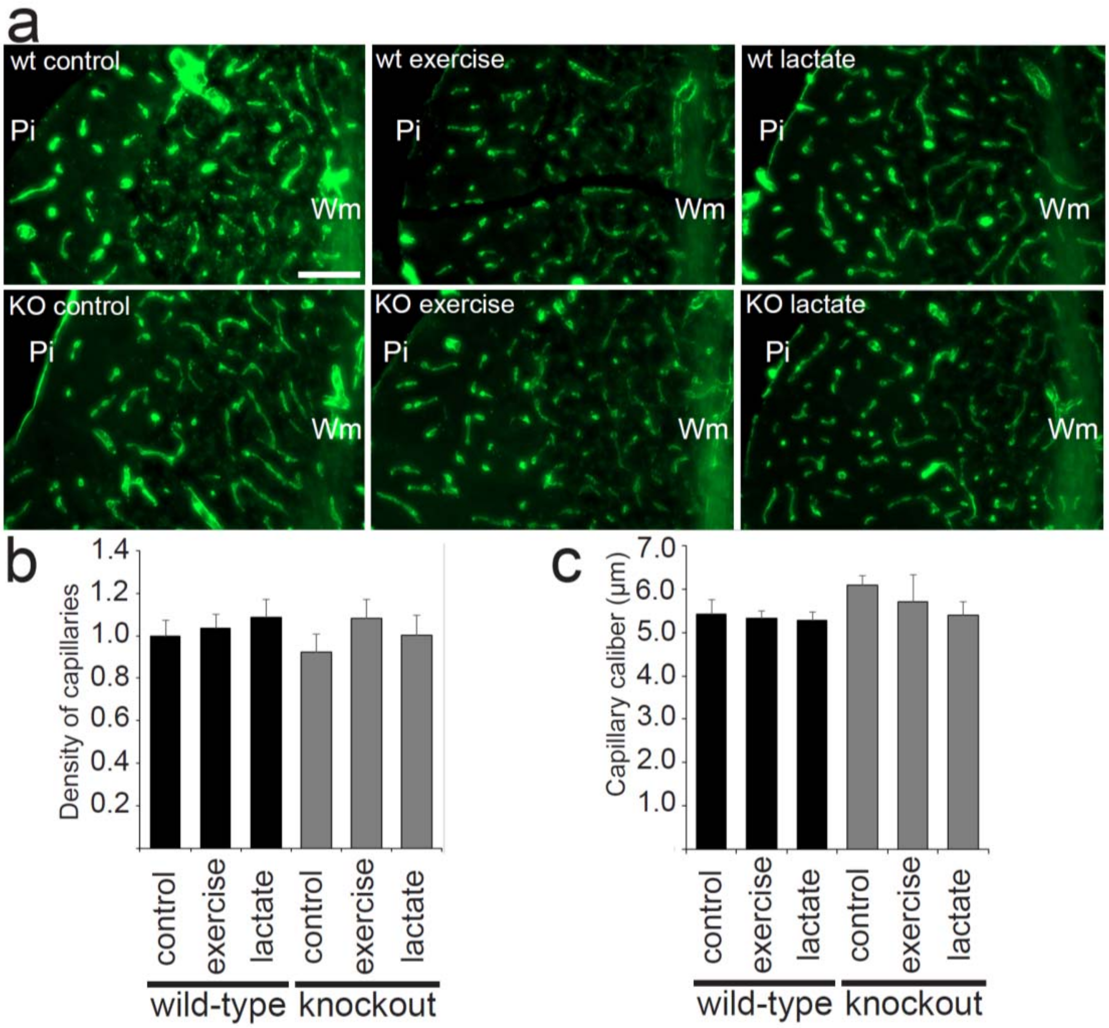

Supplementary Figure 4 | Exercise or lactate cause no change in angiogenesis in the cerebellar cortex. a, Representative images of collagen IV-labelled capillaries in the anterior lobe (folia 1-2) of the cerebellar cortex of wild-type or *Hcar1* knockout mice exposed to vehicle injections (control), treadmill exercise, or lactate injections, 5 days a week for 7 consecutive weeks. Symbols: Pia, pia mater; Wm, white matter. Scale bar: 100 µm. **b**, Quantification of vessel density (in percent of total area, normalized to wild-type control) in the cerebellar cortex. Mean ± s.e.m. of n = 5 wild-type controls, 6 wild-type exercise, 5 wild-type lactate, 5 knockout controls, 4 knockout exercise, and 6 knockout lactate mice. ANOVA, $P > 0.05$. **c**, Quantification of vessel diameter in the cerebellar cortex. Mean ± s.d. of the same animals. ANOVA, $P > 0.05$. (Same sections and analysis method as in Fig. 1b.)

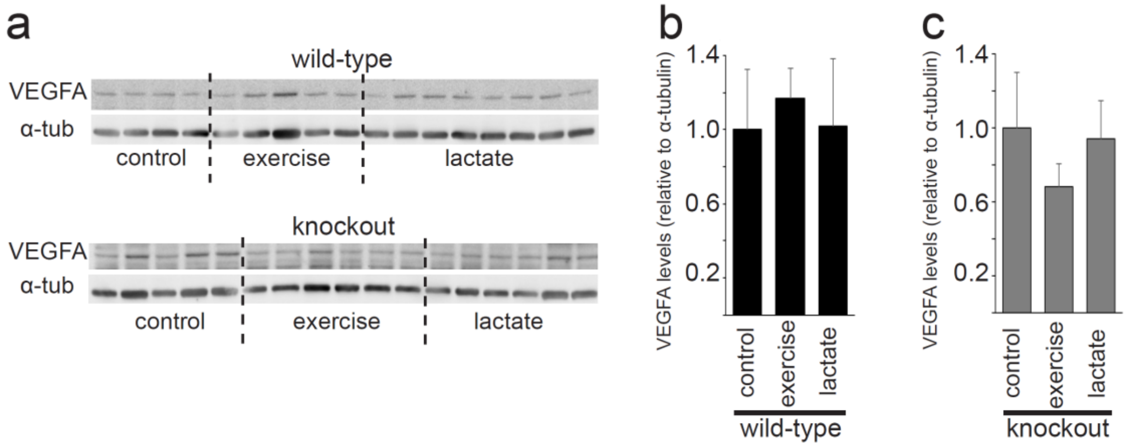

Supplementary Figure 5 | Exercise or lactate cause no significant change in VEGFA in cerebellum. a, Images of Western blots underlying analysis in b and c. (Uncropped blots presented in Supplementary Fig. 6.) **b**, Quantification of VEGFA in cerebellum of wild-type mice. Data are relative to α-tubulin (α-tub), normalized to saline injected wild-type control mice and presented as mean ± s.e.m. of n = 4 wild-type control mice, 5 wild-type exercised mice, and 8 wild-type mice treated with lactate. ANOVA, $P = 0.64$. **c**, Quantification of VEGFA in cerebellum of knockout mice. Data are relative to α-tubulin (α-tub), normalized to saline injected knockout control mice and presented as mean ± s.e.m. of n = 5 knockout control mice, 6 knockout exercised mice, and 6 knockout mice treated with lactate. ANOVA, $P = 0.06$.

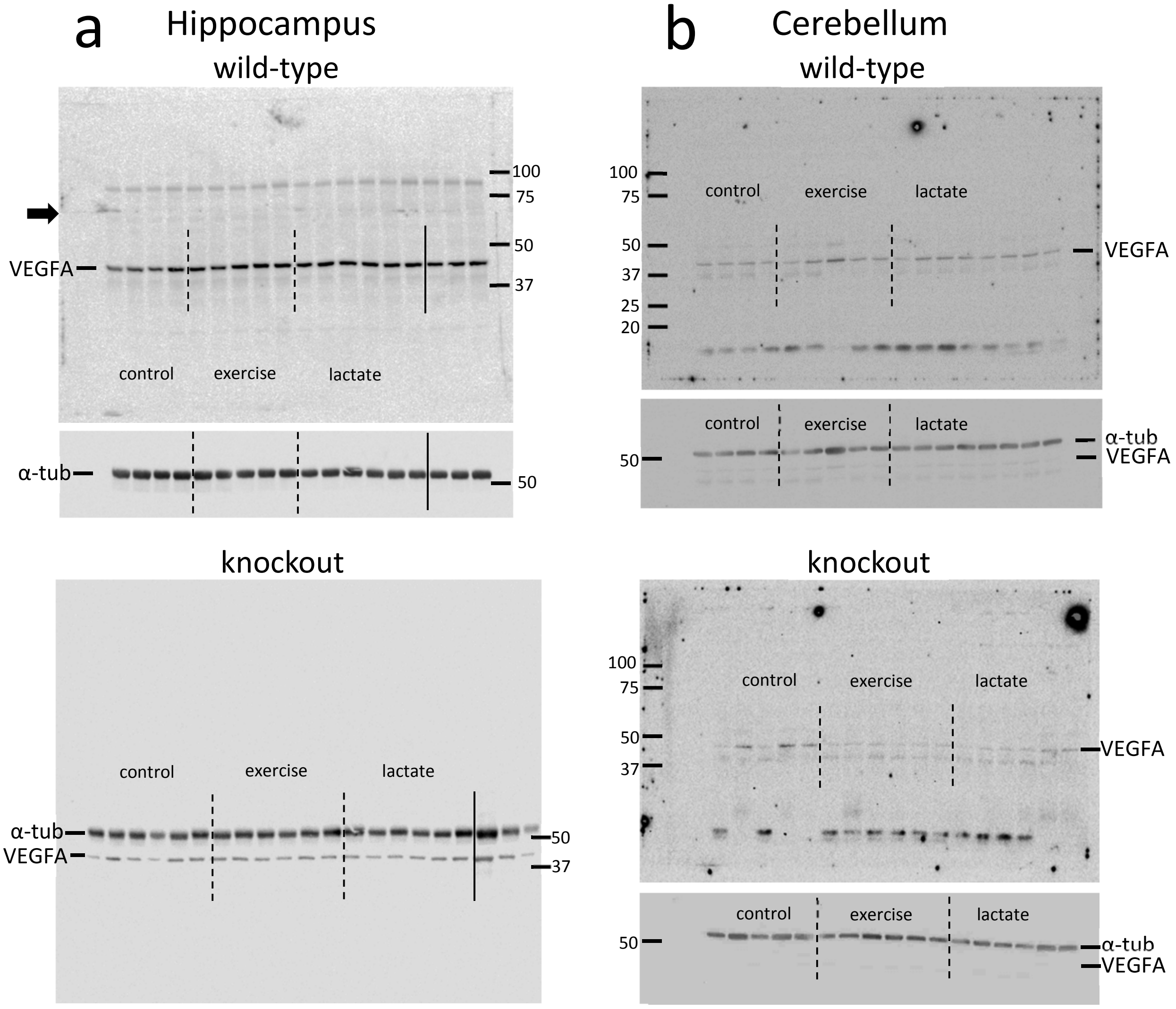

Supplementary Figure 6 | Uncropped scans of Western blots. **a,** Western blots for Fig. 1g, hippocampus. **b,** Western blots for Supplementary Fig. 5a, cerebellum. The membranes were first incubated with VEGFA antibody, then α-tubulin (α-tub) antibody (expected molecular masses VEGFA 43 kDa and α-tub 52 kDa). Among luminescence scans of different exposure times, we selected pictures in which the bands showed suitable intensities for displaying in Fig. 1g and Supplementary Fig. 5a. The scan in a, upper panel, is somewhat over-exposed, serving to show the extent of the membrane. (The upper part of the membrane was cut away, at the fat arrow, to use in an unrelated experiment; it is shown here only for completeness.) The three lanes to the right of the solid vertical lines in a are from heterozygose mice injected with saline (cropped off in Fig. 1g). In cerebellum, the VEGFA antibody showed some extraneous bands that were not seen in hippocampus. The positions of molecular mass markers are indicated, kDa.

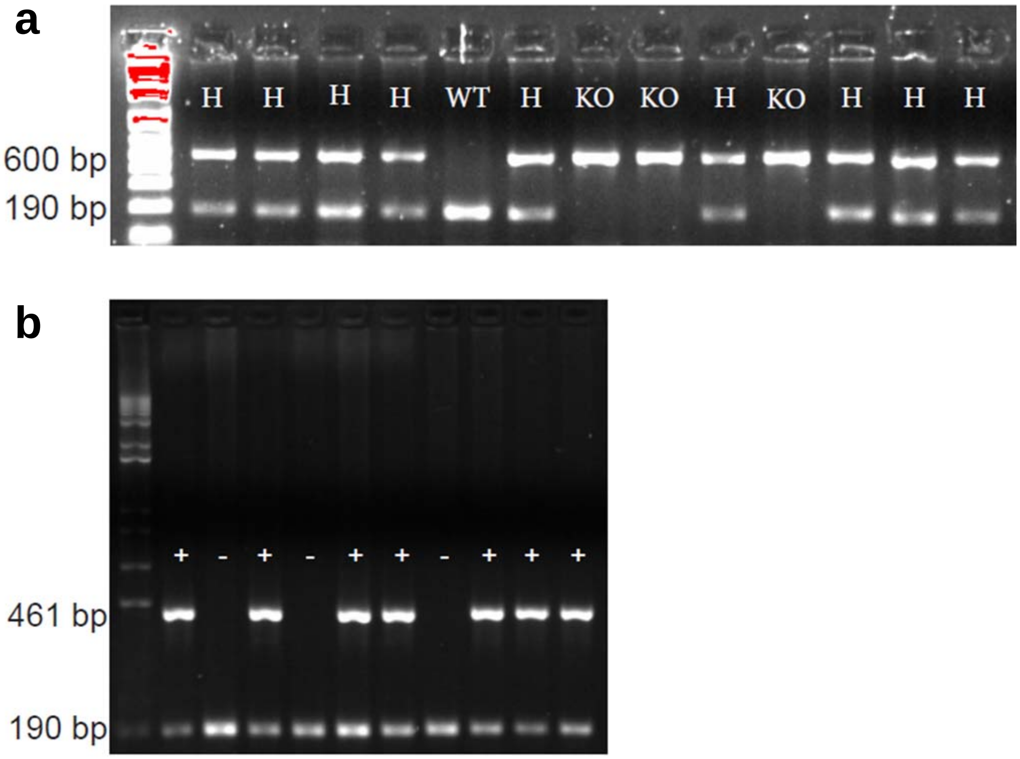

Supplementary Figure 7 | Transgenic mice, genotypes used. **a**, *Hcar1* knockout (KO) mice have the 190 bp *Hcar1* gene of the wild-type (WT) replaced by the 600 bp *lacZ* gene[2], both genes are present in heterozygous (H) mice. **b**, mRFP-HCAR1 mice (+) express both *mRFP* (461 bp) and endogenous *Hcar1* (190 bp) under control by the *HCAR1* promoter[2], wild-type mice (-) only endogenous *Hcar1*. Southern blots.

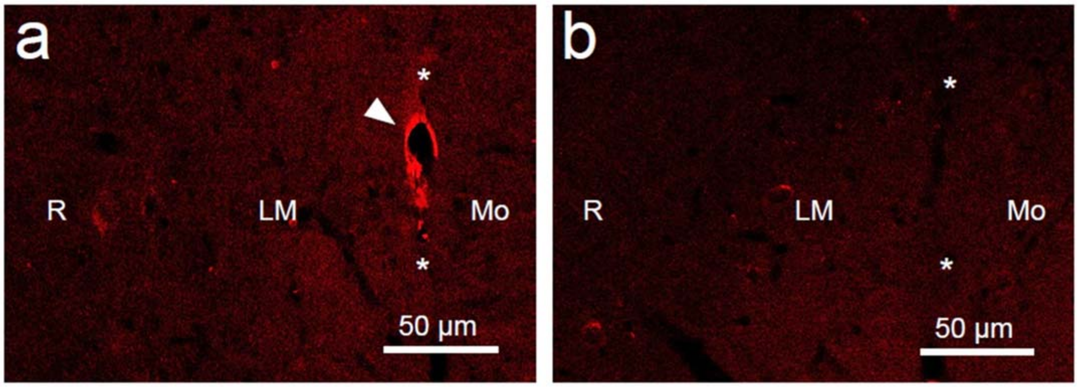

Supplementary Figure 8 | Specificity of mRFP immunofluorescence signal. **a**, mRFP-HCAR1 expressing mouse. **b**, wild-type mouse. Sections were treated with anti-mRFP antibody. Area surrounding fissura hippocampi (asterisks) is shown, with stratum radiatum (R) and stratum lacunosum-moleculare (LM) of hippocampus CA1, and the outer zone of the molecular layer of area dentata (Mo) on opposing sides of the fissure. Arrowhead (**a**) indicates mRFP-HCAR1 expressing pial cells at a blood vessel running in the fissura hippocampi (compare Fig. 3d,i). No mRFP-HCAR1 at vessels in wild-type mouse (**b**).

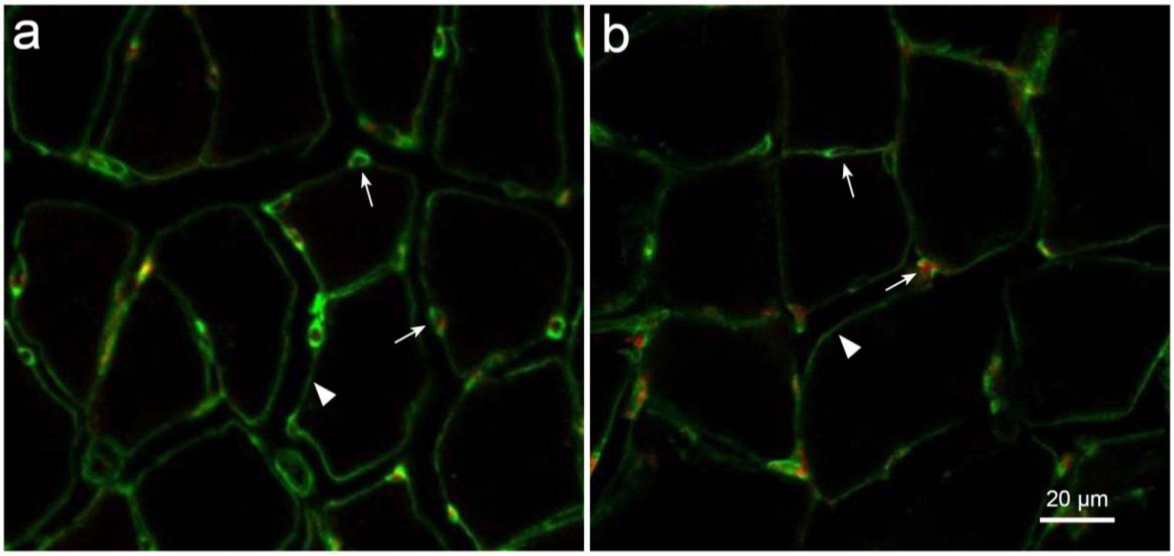

Supplementary Figure 9 | No significant HCAR1 localization in skeletal muscle. **a**, mRFP-HCAR1 reporter mouse. **b**, wild-type mouse. Soleus part of the triceps surae muscle. Basement membrane in capillaries (arrows) and around muscle fibres (arrowheads) are visualized by immunolabelling for collagen IV (green). No significant mRFP-HCAR1 signal (red); traces of red in some capillaries, in both a and b, represent autofluorescence.

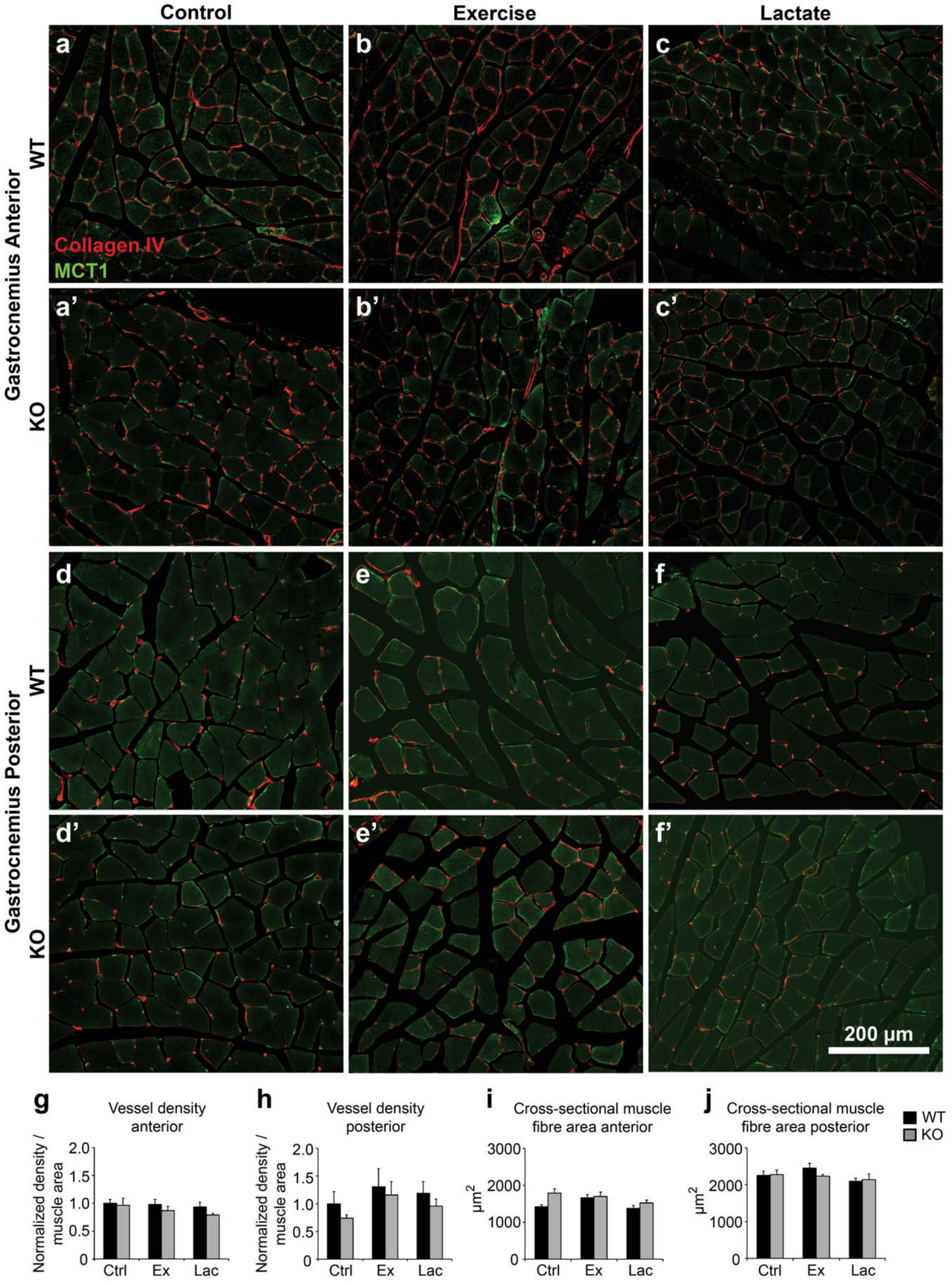
Control
Exercise
Lactate
Gastrocnemius Anterior
WT
KO
Gastrocnemius Posterior
WT
KO
a
b
c
a'
b'
c'
d
e
f
d'
e'
f'
Collagen IV
MCT1
200 μm
g
Vessel density anterior
h
Vessel density posterior
i
Cross-sectional muscle fibre area anterior
j
Cross-sectional muscle fibre area posterior
WT
KO
Normalized density / muscle area
μm²
Ctrl
Ex
Lac
2.0
1.5
1.0
0.5
0
3000
2000
1000
0

Supplementary Figure 10 | No angiogenic effect of exercise or lactate in skeletal muscle. a-f, Images of collagen IV-labelled capillaries (red) in the anterior (a-c) and posterior (d-f) gastrocnemius muscle (parts of the triceps surae muscle) of wild-type (a-f) or *Hcar1* knockout (a'-f') mice exposed to vehicle injections (control), treadmill exercise, or lactate injections, 5 days per week for 7 consecutive weeks. The sections were co-labelled for monocarboxylate transporter 1 (MCT1, green) to visualize the muscle fibres. **g-h**, Normalized density ± SEM of collagen IV-labelled capillaries associated with muscle fibres in gastrocnemius anterior (g) and posterior (h). **i-j**, Average cross-sectional area ± s.e.m. of muscle fibres in gastrocnemius anterior (i) and posterior (j). Ctrl, sedentary vehicle control; Ex, exercise; Lac, lactate. n = 5-6 mice per group. ANOVA showed no significant differences among the results, except in i, but the post hoc test showed significance only between the controls.

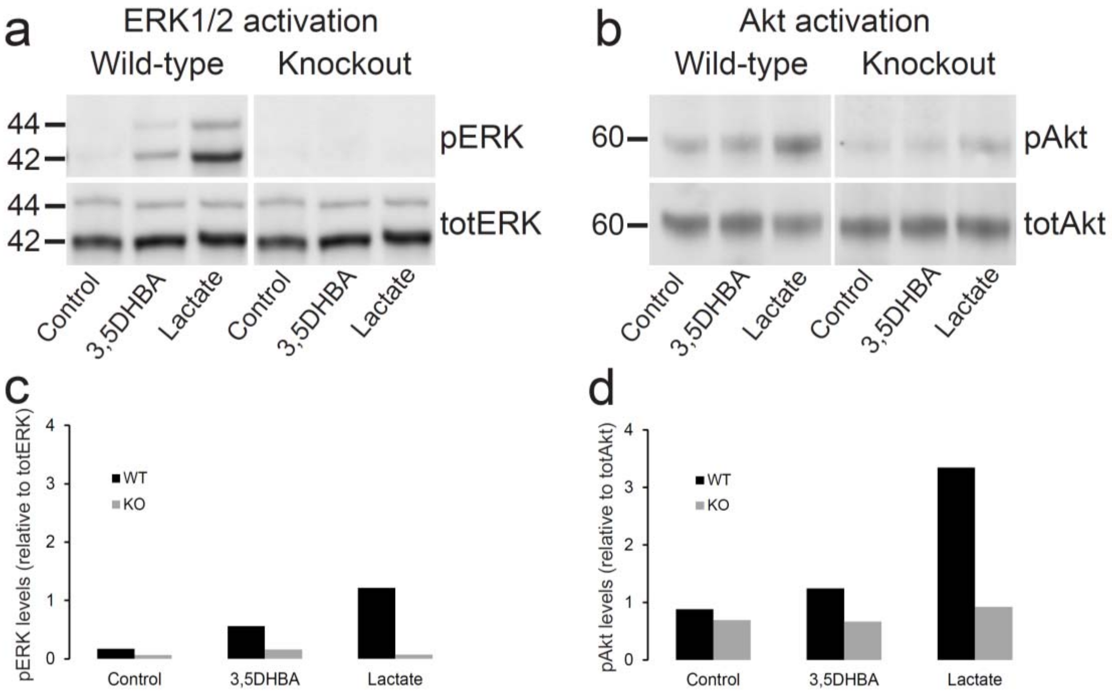

Supplementary Figure 11 | HCAR1 activates ERK1/2 and Act, which are known to induce angiogenesis through VEGFA[3].

a-b, Western blots showing HCAR1 stimulation of phosphorylation of ERK1/2 and Akt in mouse hippocampal slices. **a**, Phosphorylated ERK (pERK; upper panel) in *Hcar1* knockout and wild-type slices after exposure to Krebs buffer (Control) or Krebs buffer containing the HCAR1 selective agonist 3,5-dihydroxybenzoate (3,5DHBA) 2mM, or sodium L-lactate (Lactate) 10mM, for 90 minutes at 30°C. Both agonists induced ERK phosphorylation in wild-type mice but not in *Hcar1* knockouts. **b**, Phosphorylated Akt (pAkt; upper panel) in *Hcar1* knockout and wild-type slices as in a. Both agonists induced Akt phosphorylation in wild-types but not in *Hcar1* knockouts. Total ERK (totERK) and total Akt (totAkt) signals were largely unaffected by treatment and genotype. **c**-**d**, Quantification of Western blots as shown in a and b, expressed relative to totERK1/2 and totAkt, respectively, used as loading controls; means of two runs.

Supplementary References

1. Shepherd JK, Grewal SS, Fletcher A, Bill DJ, Dourish CT. Behavioural and pharmacological characterisation of the elevated "zero-maze" as an animal model of anxiety. *Psychopharmacology (Berl)* **116**, 56-64 (1994).

2. Ahmed K*, et al.* An autocrine lactate loop mediates insulin-dependent inhibition of lipolysis through GPR81. *Cell Metab* **11**, 311-319 (2010).

3. Wang L*, et al.* Neural progenitor cells treated with EPO induce angiogenesis through the production of VEGF. *J Cereb Blood Flow Metab* **28**, 1361-1368 (2008).

Supplementary Table 1 | *Hcar1* expression in tissues of wild-type and knockout mice

EXP.#	TISSUE	WILD-TYPE	SEM	n	KNOCKOUT	SEM	n
**1**	Hippocampus	1	0.52	5	0.00030	0.00015	5
	Liver	0.0046	0.0038	4	0.00024	0.00009	3
	Muscle	0.342	0.093	5	0.00008	0.00004	4
	Fat	75	30	4	0.0013	0.0012	2
**2**	Hippocampus	1	0.148	4	<0.008	<0.001	5
	Meninges	7.6		5*	0.08		5*

Hcar1 mRNA expression is given relative to that of the house-keeping gene, *RPS18r* (Experiment #1), or *Rpl27a* (Experiment #2), and normalized to the value for hippocampus (= 1). SYBR-green (Experiment #1) or TaqMan based detection (Experiment #2) was used.

*, Meninges from 5 mice were pooled to get enough material for analysis.

Supplementary Table 2 | Capillary area fraction and volume fraction in sensorimotor cortex

	wt vehicle	wt exercise	wt lactate	ko vehicle	ko exercise	ko lactate
**AF**	8.36±0.49	9.40±0.45	9.99±0.86	8.44±0.31	8.13±0.68	8.31±0.69

The table shows the area fraction (AF, % of tissue volume, mean ± s.d.) of capillaries in sensorimotor cortex, i.e., the 'raw data' underlying the relative values of capillary density presented for wild-type (wt) and *Hcar1* knockout (ko) mice in the main Fig. 1b (there as mean ± s.e.m.). The AF was determined blind by point-counting on images acquired from 20 µm parasagittal sections using an automated slide scanner system (see Methods). The volume fraction (VF) can be obtained from the area fraction (AF), the mean capillary diameter (dC), and the section thickness (t), through the formula VF = AF*dC/t (see Weber, B., Keller, A.L., Reichold, J. & Logothetis, N.K. The microvascular system of the striate and extrastriate visual cortex of the macaque. *Cereb. Cortex* **18**, 2318-2330 (2008), in its Appendix on stereological computations, p 2328). AF = 8.4% in wt as well as ko controls (this Table), dC = 5.8 µm (outer diameter, equal in all the experimental conditions, see legend of Fig.1), and t = 20 µm. Hence VF = 8.4%*5.8µm/20µm = **2.4%**. Thus capillaries occupy 2.4% of the volume of cortex gray matter in the control condition. (Note that the conversion factors do not change among the experimental conditions, and that conversion to 'absolute' VF values is not required for determining the relative changes in capillary density caused by exercise and lactate, displayed in Fig. 1b.)
